# General practice service use at the end-of-life before and during the COVID-19 pandemic: a population-based cohort study using primary care electronic health records

**DOI:** 10.3399/BJGPO.2023.0108

**Published:** 2024-03-20

**Authors:** Emeka Chukwusa, Stephen Barclay, Martin Gulliford, Richard Harding, Irene Higginson, Julia Verne

**Affiliations:** 1 King’s College London, Florence Nightingale Faculty of Nursing Midwifery and Palliative Care, Cicely Saunders Institute, London, UK; 2 Department of Public Health and Primary Care, University of Cambridge, Cambridge, UK; 3 Department of Population Health Sciences, King’s College London, Faculty of Life Science & Medicine, London, UK; 4 Public Health England, Bristol, UK

**Keywords:** COVID-19, general practitioners, palliative care, pandemics, primary health care, remote consultation, telephone, terminal care

## Abstract

**Background:**

Globally, the COVID-19 pandemic has caused unprecedented strain in healthcare systems, but little is known about how it affected patients requiring palliative and end-of-life care from GPs.

**Aim:**

To evaluate the impact of the pandemic on primary care service use in the last 3 months of life, including consultations and prescribing, and to identify associated factors.

**Design and setting:**

A retrospective cohort study in UK, using data from the Clinical Practice Research Datalink.

**Method:**

The study cohort included those who died between 2019 and 2020. Poisson regression models using generalised estimation equations were used to examine the association between primary care use and patient characteristics. Adjusted rate ratios (aRRs) and 95% confidence intervals (95% CIs) were estimated.

**Results:**

A total of 44 534 patients died during the study period. The pandemic period was associated with an 8.9% increase in the rate of consultations from 966.4 to 1052.9 per 1000 person-months, and 14.3% longer telephone consultation duration (from 10.1 to 11.5 minutes), with a switch from face-to-face to telephone or video consultations. The prescription of end-of-life care medications increased by 6.3%, from 1313.7 to 1396.3 per 1000 person-months. The adjusted rate ratios for consultations (aRR = 1.08, 95% CI = 1.06 to 1.10, *P*<0.001) and prescriptions (aRR 1.05: 95% CI = 1.03 to 1.07, *P*<0.001) also increased during the pandemic.

**Conclusion:**

The pandemic had a major impact on GP service use, leading to longer consultations, shifts from face-to-face to telephone or video consultations, and increased prescriptions. GP workload-related issues must be addressed urgently to ease the pressure on GPs.

## How this fits in

The pandemic has put unprecedented strain on healthcare systems across the world. To combat the spread of coronavirus, many countries authorised measures such as social distancing, facial masking, and stay-at-home orders (lockdowns). These policies altered the way health and social care services were accessed and delivered. We found that the pandemic resulted in a switch from face-to-face to telephone or video consultations, increased rate of consultations and duration of telephone consultations, and increased prescriptions of medications used in community end-of-life care. The findings highlight the challenges that GPs encountered while caring for patients towards the end of their lives.

## Background

The pandemic has subjected healthcare systems around the world to unprecedented strain and disruption, including the UK. To contain the spread of coronavirus, a series of measures including social distancing, facial masking, and stay-at home orders (lockdowns) were mandated across the UK.^1^ These measures significantly changed how health and social care services were accessed and delivered. Many aspects of GP services were affected, including the shift to virtual care from the traditional care delivery model of face-to-face consultations.^2^ The workforce and care capacity were also affected.^3^

Recent evidence from a systematic review of studies from 20 different countries showed that overall health service use declined by one-third during the pandemic.^4^ A decline in the intensity of health service use measured by consultation counts was reported in a Swiss study.^5^ In Ireland, there was a decrease in face-to-face consultations and a corresponding increase in telemedicine consultations.^6^ UK studies showed a decline in prescribing during the pandemic.^7–9^ Other studies have reported reductions in GP service use in several disease groups^10–12^ and decreased urgent referral rates for cancer.^13,14^

There is evidence that the COVID-19 pandemic exacerbated the existing or created new inequalities in health and care. For instance, people from racial and ethnic minority groups,^15^ including those with low household income, were at higher risks of COVID-19 infection and death.^16^

During the COVID-19 pandemic, palliative specialists became busier,^17^ but many community services (such as care provided by home health care workers) felt they were overlooked in the COVID-19 response.^18^ There is little research on how support from GPs for patients needing palliative and end-of-life care was affected by the COVID-19 pandemic and measures such as lockdowns. This study aimed to evaluate the impact of the pandemic on primary care service use in the last 3 months of life, including consultations and prescribing, and to identify factors associated with use before and during the pandemic.

## Method

### Design and data sources

We conducted a retrospective cohort population-based study in UK primary care, using data from the CPRD Gold database build May 2021. The CPRD is a database containing anonymised records of primary care activity, providing one of the world’s largest longitudinal datasets of primary care.^19^ The CPRD Gold database is broadly representative of the UK population in terms of patients’ demographic characteristics.^19^ The study protocol^20^ was approved by the CPRD Independent Scientific Advisory Committee (reference: 20_131R).

### Population

The study population included all patients registered in up-to-standard (UTS) GP practices in the UK who died in 2019 and 2020. UTS is a practice-based quality marker used by the CPRD to adjudge periods of quality data recording.^19^ Our cohort included patients in the final 3 months of life and who had been continuously registered for at least 1 year.

### Outcome

The main study outcome was GP service use, expressed as counts of consultations and prescriptions in the final 3 months of life. Patients who died 3 months after the end of 2020 (that is, up until the month of March 2021) were included to ensure that all GP contacts in their final 3 months of life were captured. Consultations were categorised according to the mode of delivery: telephone, face-to-face, video, and others (including administration-related and email correspondence between GPs and patients). Consultation categories were derived from the ‘constype’ (consultation type) variable and medical diagnostic codes used in a previous study.^21^ Video consultations were identified through wildcard and keyword searches of the clinical files, and the CPRD code browser software. For statistical modelling, video and telephone consultations were combined. We used a list of medications commonly used for symptom control in community end-of-life care identified in a previous study,^22^ from which we used a data-driven approach to identify the top five medications commonly prescribed during the study period (oxycodone, midazolam, morphine, codeine, and paracetamol). Other medications were categorised as ‘others’.

### Explanatory variables

To describe changes in primary care use, the data were stratified into the pre-pandemic (2019) and pandemic (2020) periods. Given the start of the pandemic was in March 2020, we restricted the study period to the months of March to December in both years to ensure an equal numbers of months in both pandemic and pre-pandemic periods. Therefore, the comparative periods were 1 March 2019 to 31 December 2019 (pre-pandemic) and 1 March 2020 to 31 December 2020 (pandemic). For visualisation of rates, we included data from the months of January to December to capture trends in service use before the introduction of the first national lockdown in March 2020.

Explanatory variables included: age group; sex; marital status; practice index for multiple deprivation (IMD) (practice location urban/rural; region of UK); number of comorbidities in period before death; care home residency; and identified palliative care need. Some of these variables were derived from clinical codes used in previous studies.^23–25^

### Analysis

Data were described using counts and percentages for categorical variables. Continuous variables were described using means and standard deviation (SD). Changes in the patterns of GP service use between the pre-pandemic and pandemic period were quantified with percentage changes. Time trends in monthly rates of service use were plotted against months and visualised with line graphs. Rates were derived from the aggregate monthly counts of service use (numerator) divided by the respective person-times (person-months) at risk (denominator). Mean duration or length of consultations (in minutes) was calculated for each month, and variation was plotted between the pandemic and pre-pandemic levels. In line with the approach used in previous studies,^26,27^ anomalously high values of consultation durations (that is, >60 minutes) were rounded down to 60 minutes, and those with values of 0 were rounded up to 0.5 minutes, as it was unlikely that duration of consultations would last less than 1 minute or longer than 1 hour.

Factors associated with GP service use were modelled using Poisson models fitted using the method of generalised estimating equations, to examine the association between indicators of GP service use (prescriptions and consultations) and patients’ sociodemographic characteristics. The encrypted unique GP practice identifier (practice ID) was included to account for clustering of service use within each GP practice, on the assumption that patients clustered within the same GP practice had similar patterns of service use. The natural logarithm of the patient’s person-years at risk was included as an offset variable in each model. We conducted sensitivity analysis by using cohorts of patients in the final 6 months of life. The models for sensitivity analysis were fitted with negative binomial regression, owing to convergence and overdispersion. The strength of the associations identified was described using multiple adjusted rate ratios (aRRs) and 95% confidence intervals (95% CIs). All statistical analyses, including data visualisations, were completed with R version 4.1.2.

## Results

### Patients’ sociodemographic characteristics stratified by period

The study cohort comprised 44 534 patients (mean age = 77 years, SD = 14.7–14.4) from 365 GP practices who died during the study period (Table 1).

**Table 1.** Patients clinical and sociodemographic characteristics before and during the pandemic

	Level	Overall	2019 (March–Dec)	2020 (March–Dec)
*N*	Overall	44 534	20 679	23 855
**Age, mean (SD)**		77.2 (14.6)	77.1 (14.7)	77.3 (14.4)
**Age group, *n* (%)**	<50	2479 (5.6)	1162 (5.6)	1317 (5.5)
	50–59	3173 (7.1)	1472 (7.1)	1701 (7.1)
	60–69	5905 (13.3)	2793 (13.5)	3112 (13.0)
	70–79	11 207 (25.2)	5108 (24.7)	6099 (25.6)
	≥80	21 770 (48.9)	10 144 (49.1)	11 626 (48.7)
**Sex, *n* (%)**	Female	22 251 (50.0)	10 375 (50.2)	11 876 (49.8)
	Male	22 283 (50.0)	10 304 (49.8)	11 979 (50.2)
**Marital status, *n* (%)**	Civil partnership/Co-habiting/Engaged/Stable relationship/Married/Remarried	8133 (18.3)	3831 (18.5)	4302 (18.0)
	Divorced/Separated/Dissolved/Widowed	2069 (4.6)	987 (4.8)	1082 (4.5)
	Single	4265 (9.6)	1995 (9.6)	2270 (9.5)
	Unknown/Not stated	30 067 (67.5)	13 866 (67.1)	16 201 (67.9)
**Comorbidities, *n* (%)**	0	4159 (9.3)	1843 (8.9)	2316 (9.7)
	1	7343 (16.5)	3399 (16.4)	3944 (16.5)
	2	7821 (17.6)	3698 (17.9)	4123 (17.3)
	3	7280 (16.3)	3403 (16.5)	3877 (16.3)
	4	6147 (13.8)	2875 (13.9)	3272 (13.7)
	≥5	11 771 (26.4)	5456 (26.4)	6315 (26.5)
	Missing	13 (0.0)	5 (0.0)	8 (0.0)
**Care home residency status, *n* (%)**	False	38 790 (87.1)	18 050 (87.3)	20 740 (86.9)
	True	5731 (12.9)	2624 (12.7)	3107 (13.0)
	Missing	13 (0.0)	5 (0.0)	8 (0.0)
**Palliative care recognised, *n* (%)**	No	26 493 (59.5)	12 338 (59.7)	14 155 (59.3)
	Yes	18 028 (40.5)	8336 (40.3)	9692 (40.6)
	Missing	13 (0.0)	5 (0.0)	8 (0.0)
**Settlement, *n* (%)**	Rural	6226 (14.0)	2936 (14.2)	3290 (13.8)
	Urban	38 308 (86.0)	17 743 (85.8)	20 565 (86.2)
**IMD, *n* (%)**	1 (least deprived)	7457 (16.7)	3508 (17.0)	3949 (16.6)
	2	7089 (15.9)	3307 (16.0)	3782 (15.9)
	3	8674 (19.5)	4089 (19.8)	4585 (19.2)
	4	11 178 (25.1)	5158 (24.9)	6020 (25.2)
	5	10 136 (22.8)	4617 (22.3)	5519 (23.1)
**Region, *n* (%)**	England	5302 (11.9)	2316 (11.2)	2986 (12.5)
	Northern Ireland	4077 (9.2)	1912 (9.2)	2165 (9.1)
	Scotland	20 644 (46.4)	9788 (47.3)	10 856 (45.5)
	Wales	14 511 (32.6)	6663 (32.2)	7848 (32.9)

IMD = index of multiple deprivation. SD = standard deviation.

### Changes in rates and duration of GP consultations

A total of 168 236 consultations took place during the final 3 months of life (Table 2).

**Table 2.** Changes in GP consultations and prescribing in the last 3 months of life

	Pre-pandemic	Pandemic	Change, %
**GP consultations**			
Counts	74 905	93 331	24.6
Total person-months	67 537.7	77 756.6	15.1
Rate per 1000 person-months	966.4	1052.9	8.9
Number of face-to-face consultations	36 243	23 090	-36.3
Face-to-face, rate per 1000 person-months	536.6	297.0	-44.7
Telephone (including video) consultations, *n*	29 027	58 777	102.5
Telephone/video, rate per 1000 person-months	429.8	755.9	75.9
**Mean duration of consultations, minutes^a^**	**10.1**	**11.5**	**14.3**
**GP prescription**			
Total (all prescription)	88 723	108 572	22.4
Total person-months	67 537.7	77 756.6	15.1
Rate per 1000 person-months	1313.7	1396.3	6.3
Total (based on top five drugs)	55 183	69 336	25.6
Codeine phosphate/paracetamol, rate per 1000 person-months	72.9	77.3	6.0
Midazolam hydrochloride, rate per 1000 person-months	104.4	124.6	19.4
Morphine sulphate, rate per 1000 person-months	101.2	114.8	13.4
Oxycodone hydrochloride, rate per 1000 person-months	266.3	278.1	4.5
Paracetamol, rate per 1000 person-months	272.3	297	9.1

a Excluding administration-related consultations (others).

**Figure 1.**
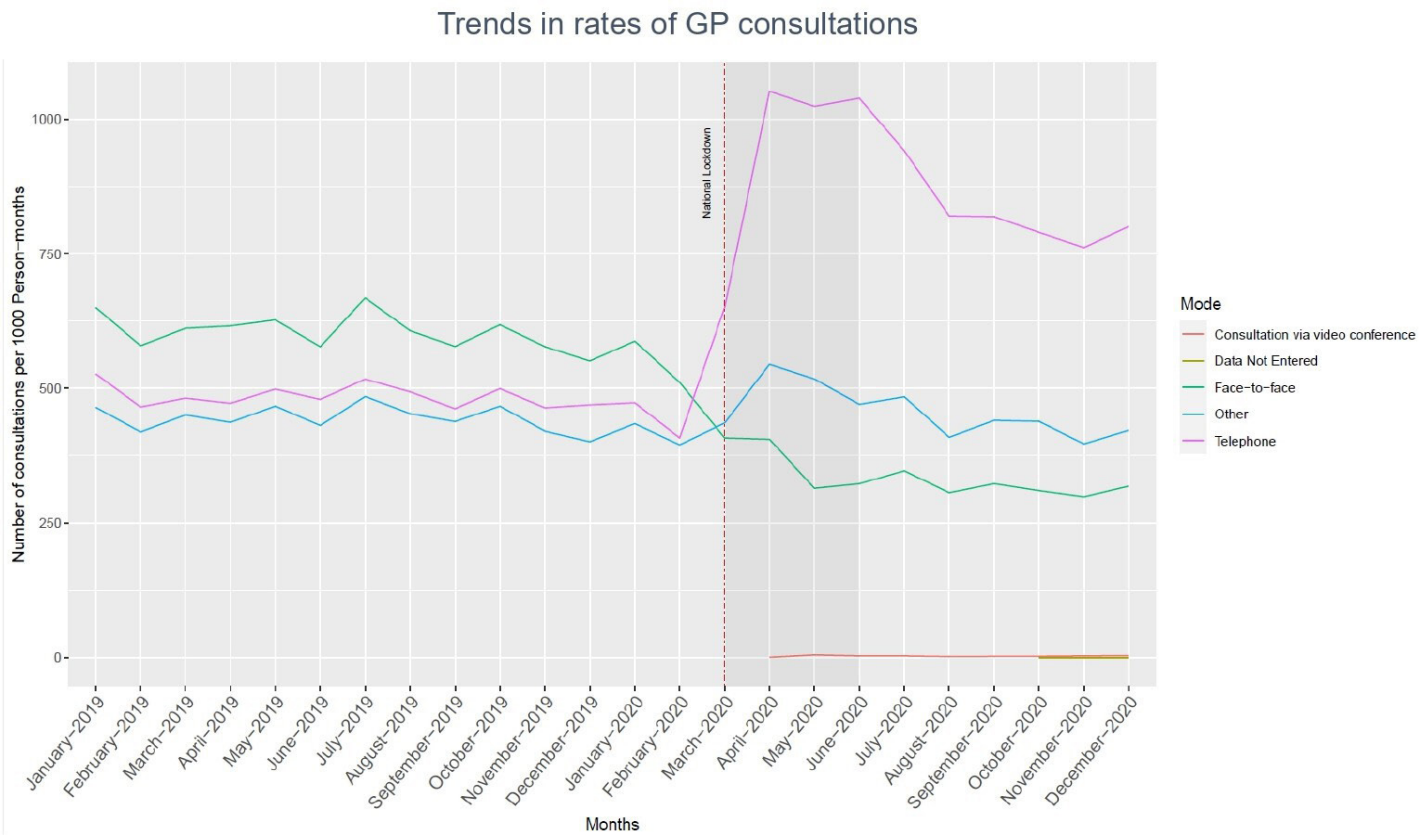
Trends in rates of GP consultations. *A non colour-dependent version of this image is available in the supplementary material.*

GP consultation rates increased during the pandemic by 8.9%, from 966.4 to 1052.9 per 1000 person-months. The rate of telephone consultations (including video consultation) increased by 75.9%, from 429.8 to 755.9 per 1,000 person-months. At the same time, there was a corresponding decrease in face-to-face consultations by 44.7%, from 536.6 to 296.95 per 1000 person-months. The mode of GP consultations changed from usual face-to-face to telephone or video consults, coinciding with the announcement of the first national lockdown in March 2020 (Figure 1).

**Figure 2.**
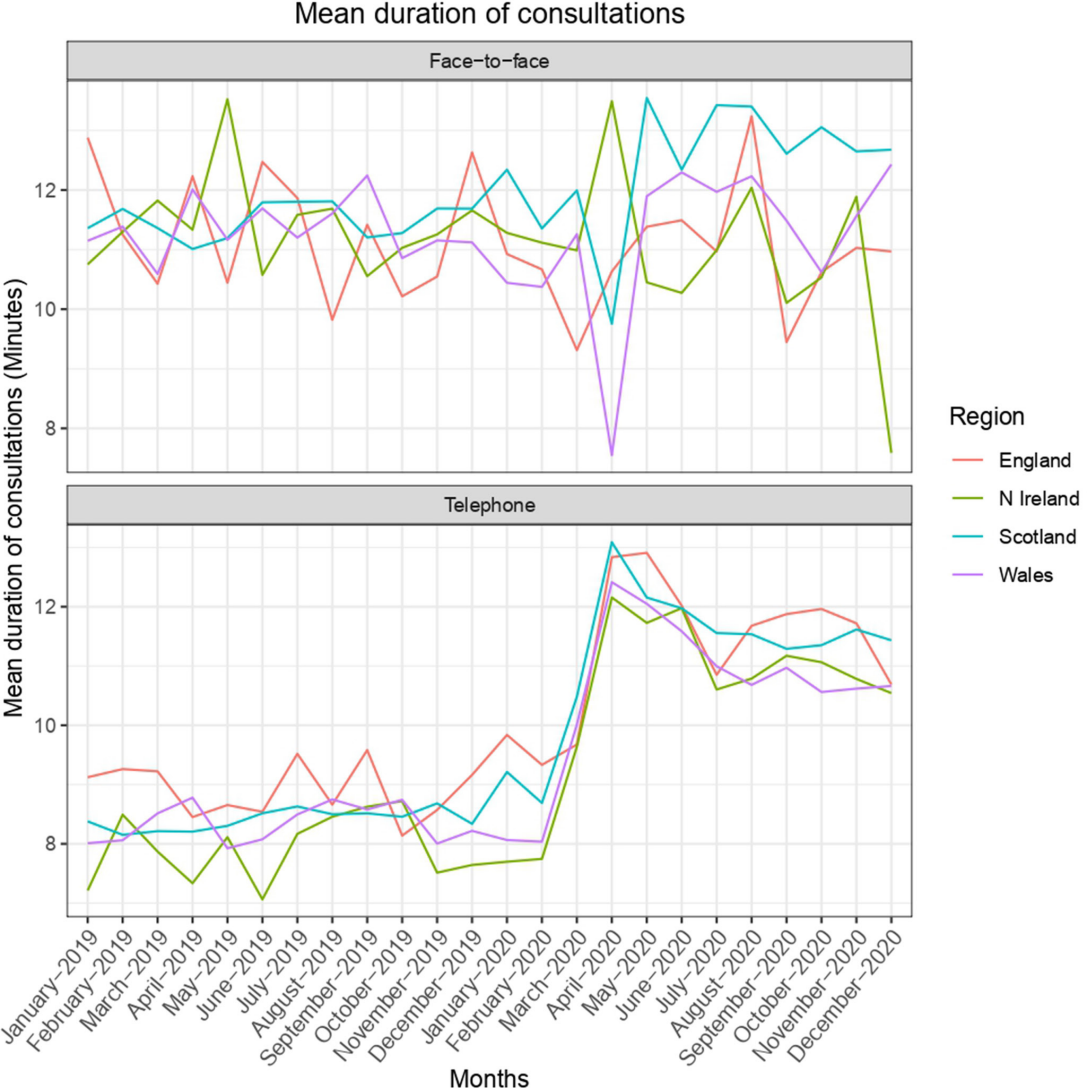
Mean duration of consultations. *A non colour-dependent version of this image is available in the supplementary material.*

The mean duration of consultations (face-to-face and telephone) before national lockdown was 10.1 minutes; this rose by 14.3% to 11.5 minutes during the pandemic (Table 2, Figure 2).

### Changes in prescriptions before and during the pandemic

The rate of prescriptions increased by 6.3%, from 1313.7 to 1396.3 per 1000 person-months (Table 2), and the rate of the top five prescribed end-of-life care medications also increased (ranging from 4.1% to 19.4%). Morphine and midazolam had the highest increases, rising by 13.4% and 19.4%, respectively.

### Factors associated with GP service use in last 3 months of life

**Figure 3.**
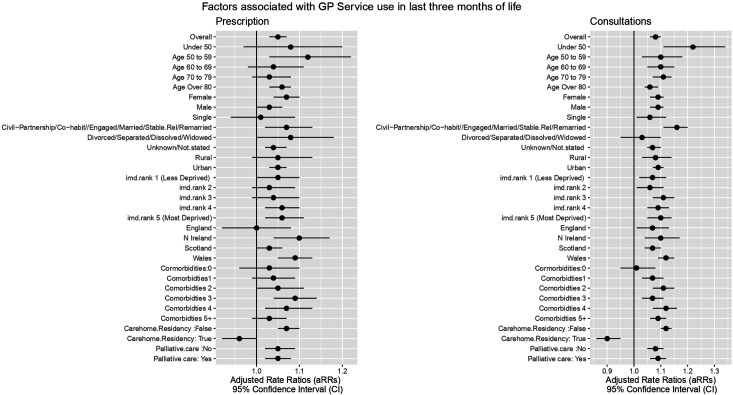
Factors associated with GP service use in the last 3 months of life.

The aRR for consultations (comprising face-to-face and telephone consultations) (Figure 3 and Supplementary Table S1) showed an overall increase (aRR = 1.08, 95% CI = 1.06 to 1.10). Being a resident of a care home was associated with fewer GP consultations (aRR = 0.90, 95% CI = 0.86 to 0.95, *P*<0.001).

The aRRs showed an overall increase (Figure 3) in prescriptions during the pandemic (aRR = 1.05, 95% CI 1.03 to 1.07, *P*<0.001). A dose response increase was observed between comorbidities and prescribing in patients with up to three comorbid conditions (aRR = 1.03 to 1.09). Being a resident of a care home was associated with fewer prescriptions during the pandemic compared with the pre-pandemic period (aRR = 0.96, 95% CI = 0.96 to 1.00, *P*=0.04). Results of sensitivity analyses based on GP service use in the final 6 months of life (Supplementary Table S1) were similar to the results obtained in the main analysis.

## Discussion

### Summary

This population-based cohort study showed an increase in GP consultations and prescriptions in the final 3 months of life , after adjusting for patients’ sociodemographic characteristics. The rate and duration of telephone consultations rose substantially after the start of the pandemic in March 2020. These changes in the patterns of service use coincided with the introduction of national lockdown in March 2020, and are consistent with findings from previous studies.^4,28,29^ These findings suggest a pandemic-related increase in workload for GPs caring for patients in the final 3 months of life.

### Strengths and limitations

This is the first study to use routinely collected electronic health records from the CPRD database to examine the impact of the pandemic on primary care use for patients approaching the end of life. A strength of this study is the use of a national dataset providing population-based data at individual patient level, broadly representative of patients’ sociodemographic characteristics in terms of age and sex.^19^

The study has several limitations. We used data from the CPRD Gold database, which collects data from practices using Vision software. Over recent years, the number of Vision practices contributing to the CPRD has decreased, and fewer practices contributed to the database during the study period. To ensure that the findings were not skewed by this changing practice denominator, we only included data from GP practices that contributed to the database throughout the study period, leading to fewer practices and patients for the analysis. Second, because many Scottish general practices have continued to use Vision software, Scottish patients and practices were overrepresented in the sample, which could limit the generalisability of the findings to other UK nations. Third, the rapid shift from face-to-face to virtual consultations may have influenced GP coding behaviour, particularly the accuracy of recording consultation types. However, we were able to cross check records of consultation categories with medical diagnostic codes from the clinical file and the CPRD browser software.

Fourth, we excluded ethnicity in the analysis due to the large proportion of missing data; this could be usefully addressed by the CPRD quality team. Fifth, we lacked information on variables including cause of death, disease severity, and reasons for patient consultations or prescriptions. Nor do we know whether medications prescribed were dispensed or taken by the patient. The inclusion of these key variables in future studies would provide further insights on how the pandemic has affected various diseases and ethnic groups. Sixth, the limited number of video consultations in the dataset meant that they were combined with telephone consultations, as they are both remote. It is important for future studies to treat both differently. Finally, we used data from the first wave of the pandemic, 2019 and 2020. The current situation of primary care service use has changed remarkably since 2020. It is important that future studies use more recent data to confirm the findings of this study.

### Comparison with existing literature

We found a significant decrease in face-to-face consultations and a corresponding increase in telephone consultations. Our findings of an overall increase in GP consultations are in contrast with previous studies, which showed an overall decline in health service use during the pandemic.^4,5^ These changes could be due to differences in population cohort, as our study focused on patients in the last 3 months of life.

The duration of telephone consultations for patients in the final 3 months of life increased during the pandemic. GPs spent longer with patients on the telephone; this may be to compensate for decreased face-to-face contact and/or because patients presented with more complex problems, requiring longer consultations.^26^ One modelling study found that digital consultations are likely to increase GP workload by up to 31%,^30^ although their duration is shorter than ordinary consultations. A recent online survey evaluating the experience and perspectives of 559 GPs and community nurses reported an increased workload and emotional toll during the first wave of the pandemic.^31^ Our data support these findings, and highlight a pressing need to address these GP workload issues arising from longer consultations during the pandemic.

Video consultation rates were low during the pandemic, comprising <1% of all consultations. This is in keeping with a recent qualitative survey of GPs that found they rarely used video consultations during the pandemic, reporting that most consultations could be done effectively by telephone.^32^ Video consultations require a lot of planning and an internet connection. The low rate of video consultation needs further investigation. Video consultation is very useful for certain patient groups, especially patients with language barriers or children. However, it is important to note that reliance on video consultations can exacerbate ‘the digital divide’ or ‘telehealth inequality’, especially in rural areas with poor internet connectivity.^33^ Diversifying consultation modes and adopting a blended model of delivery,^34^ comprising both home visits and virtual consultations, might reduce access difficulties and help meet patients’ specific needs in any future pandemic. Further guidance for professionals on optimising the use of video consultations might help widen future adoption.^35^

We found a substantial rise in prescriptions of end-of-life care medications. Midazolam, a benzodiazepine used for anxiety and agitation, showed the highest increase. Similar increases in the prescription have been reported in a previous study.^36^ We found that being a resident of a care home was associated with decreased prescription during the pandemic. This decrease might be due to national guidance issued on 28 April 2020, permitting the repurposing and reuse of end-of-life care medications in care homes, amid concern that shortages could arise.^37,38^

### Implications for research and practice

Overall, the findings of this study have implications for future pandemic or natural disaster planning. As this study has shown, the COVID-19 pandemic proved that technology in terms of online consultations can be rapidly deployed and used to good effect. For example, the use of video consultations and teleconferencing has provided primary care with a new method of contacting patients, especially in today’s practice environment of long waiting times and pressure on services. It allows GPs and other members of the primary care team to be in contact with more patients in a limited time slot. In addition, other changes, including the use of electronic prescriptions, obviate the need to visit pharmacies, especially for those with limited mobility. It is important to highlight that telephone appointments may not be effective if patients have symptoms such as a rash, a lump, or conditions that may be difficult to photograph. In such situations, it may be necessary to augment telephone calls with video calls.

The pandemic had a major impact on GP services for patients in the final 3 months of life. It resulted in a switch from face-to-face to telephone and virtual consultations, increased duration of telephone consultations, and increased prescriptions of medications used in community end-of-life care. The findings provide further evidence of the pressures faced by GPs in caring for patients at the end of life during the pandemic. GP workload issues need to be addressed urgently. These unprecedented developments may have accelerated the transition to digital services, and may inform future health care models; however, prospective studies using appropriate qualitative data are needed to assess the impact of these developments on patients' reported outcomes, particularly the quality of care received.

In addition, while the CPRD dataset is a valuable and significant resource for research, there are some issues with data completeness, quality, and GP coding that must be addressed to optimise the safe use of anonymised or de-identified data for health service research.

## Data

The dataset relied on in this article is available from CPRD, subject to protocol approval.by the Independent Scientific Advisory Committee of the Medicines and Healthcare Products Regulatory Agency (ISAC).
